# Evaluation of Four Different Analytical Tools to Determine the Regional Origin of *Gastrodia elata* and *Rehmannia glutinosa* on the Basis of Metabolomics Study

**DOI:** 10.3390/molecules19056294

**Published:** 2014-05-16

**Authors:** Dong-Kyu Lee, Dong Kyu Lim, Jung A. Um, Chang Ju Lim, Ji Yeon Hong, Young A Yoon, Yeonsuk Ryu, Hyo Jin Kim, Hi Jae Cho, Jeong Hill Park, Young Bae Seo, Kyunga Kim, Johan Lim, Sung Won Kwon, Jeongmi Lee

**Affiliations:** 1 College of Pharmacy, Seoul National University, Seoul 151-742, Korea; E-Mails: dongqchicken@snu.ac.kr (D.-K.L.); ddongq1989@snu.ac.kr (D.K.L.); jafirst11@snu.ac.kr (J.A.U.); limcj85@snu.ac.kr (C.J.L.); vastgy88@snu.ac.kr (J.Y.H.); hillpark@snu.ac.kr (J.H.P.); 2 School of Pharmacy, Sungkyunkwan University, Suwon 440-746, Korea; E-Mails: duddk1987@nate.com (Y.A.Y.); ryu.yeonsuk@gmail.com (Y.R.); 3 College of Pharmacy, Dongduk Women’s University, Seoul 136-714, Korea; E-Mail: hyojkim@dongduk.ac.kr; 4 Korea Promotion Institute for Traditional Medicine Industry, Gyeongsan 712-260, Korea; E-Mail: choze@naver.com; 5 Department of Herbology, College of Oriental Medicine, Daejeon University, Daejeon 300-716, Korea; E-Mail: genin@dju.kr; 6 Department of Statistics, Sookmyung Women’s University, Seoul 140-742, Korea; E-Mail: kyunga@sookmyung.ac.kr; 7 Department of Statistics, Seoul National University, Seoul 151-742, Korea; E-Mail: johanlim@snu.ac.kr

**Keywords:** traditional medicine, *Gastrodia elata*, *Rehmannia glutinosa*, plant metabolomics, origin discrimination, multivariate statistical analysis

## Abstract

Chemical profiles of medicinal plants could be dissimilar depending on the cultivation environments, which may influence their therapeutic efficacy. Accordingly, the regional origin of the medicinal plants should be authenticated for correct evaluation of their medicinal and market values. Metabolomics has been found very useful for discriminating the origin of many plants. Choosing the adequate analytical tool can be an essential procedure because different chemical profiles with different detection ranges will be produced according to the choice. In this study, four analytical tools, Fourier transform near‑infrared spectroscopy (FT-NIR), ^1^H-nuclear magnetic resonance spectroscopy (^1^H‑NMR), liquid chromatography-mass spectrometry (LC-MS), and gas chromatography-mass spectroscopy (GC-MS) were applied in parallel to the same samples of two popular medicinal plants (*Gastrodia elata* and *Rehmannia glutinosa*) cultivated either in Korea or China. The classification abilities of four discriminant models for each plant were evaluated based on the misclassification rate and Q^2^ obtained from principal component analysis (PCA) and orthogonal projection to latent structures-discriminant analysis (OPLS‑DA), respectively. ^1^H-NMR and LC-MS, which were the best techniques for *G. elata* and *R. glutinosa*, respectively, were generally preferable for origin discrimination over the others. Reasoned by integrating all the results, ^1^H-NMR is the most prominent technique for discriminating the origins of two plants. Nonetheless, this study suggests that preliminary screening is essential to determine the most suitable analytical tool and statistical method, which will ensure the dependability of metabolomics-based discrimination.

## 1. Introduction

Herbal medicines have been traditionally used to treat patients [1]. They contain a variety of natural pharmacologically-active compounds, which often have synergistic or complex remedial effects with fewer side effects than synthetic drugs [2,3,4]. Medicinal plants, which are the major source of Traditional Chinese Medicines (TCMs), are mainly produced in Asian countries, including Korea and China. Though medicinal plants of identical species may be used for the same healing purposes, their chemical profiles could differ because of differences in their cultivation environments. A great deal of literature has reported the effects of climate, pesticides, fertilizer, and abiotic stress on plant metabolism [5,6,7,8]. Changes in the chemical composition of plants may influence their therapeutic efficacy, which in turn may affect their quality. Therefore, the regional origin of medicinal plants should be confirmed to evaluate their medicinal and market value.

Currently, medicinal plant species and origins are authenticated largely by evaluating their phenotype. Though this method is simple, rapid, and requires only an expert’s knowledge and experience, it lacks the objectivity of scientific testing, and, therefore, it is not appropriate for quality control of medicinal plants. Moreover, phenotype evaluation is practically impossible when morphological features of the plants are destroyed by processing, such as grinding [9]. Metabolomic approaches can overcome these limitations and have been suggested to detect metabolic differences arising from genetic or environmental effects.

Metabolomic profiles have been obtained by various analytical techniques including fourier transform near-infrared (FT-NIR) spectroscopy [10], ^1^H-nuclear magnetic resonance (^1^H-NMR) spectroscopy [11], liquid chromatography-mass spectrometry (LC-MS) [12], and gas chromatography-mass spectroscopy (GC-MS) [13]. FT-NIR allows for the rapid and non-destructive analysis of the metabolome, though quantitative data at the compound level is difficult to obtain [14]. NMR ensures reproducible and rapid analysis; however, its low sensitivity prevents quantifying metabolites at low concentrations [15]. MS can be advantageous over ^1^H-NMR and FT-NIR given that it provides higher sensitivity and broader detection range despite its relatively poor reproducibility [16]. Accordingly, researchers need to select a suitable approach depending on their objective. For metabolomics-based authentication of medicinal plant origins, choosing the adequate analytical tool is essential because the choice will affect the chemical profile and detection range [17]. In this study, four analytical tools, FT-NIR, LC-MS, GC-MS, and ^1^H-NMR, were used in parallel to determine the regional origin (Korea *vs.* China) of two traditional medicinal plants, *Gastrodia elata* and *Rehmannia glutinosa*, which are commonly used in Asian countries. *G. elata* is known to possess hypoglycemic [18], hemostatic and immunologic [19] activities and *R. glutinosa* is known for its anti-convulsive [20], anti-inflammatory and anti-angiogenic effects [21]. Using eight discriminant models derived from the metabolic profiling and two multivariate statistical analyses, we have attempted to determine the best approach for each species.

## 2. Results and Discussion

### 2.1. Metabolomic Profiling and Putative Identification of Metabolites with Four Analytical Approaches

Experimental conditions including sample preparation and instrumental setting were optimized for each analytical approach based on literatures and our own experiments. As a result, metabolomic profiles were successfully obtained for all tested samples using four analytical approaches: FT-NIR (Supplementary Figure S1), ^1^H-NMR (Supplementary Figure S2), LC-MS (Supplementary Figure S3), and GC-MS (Supplementary Figure S4). As reviewed previously [14,16,17], each analytical approach had advantages and disadvantages. In the FT-NIR analysis, which has been suggested as a first-round screening technique for metabolic fingerprinting [22], sample preparation was convenient, but identifying spectral peaks arising from complex functional groups was extremely difficult. Relatively, it is possible for LC-MS and GC-MS to separate and identify the peaks of a large number of metabolites.

For the LC-MS analysis, inconsistent retention times and fragment patterns in the spectra have prevented the development of a public database; therefore, the use of standard compounds was necessary to identify metabolites. In addition, assigning secondary metabolites requires separating compounds at baseline, which can be time-consuming. Although a number of secondary metabolites in *Schisandra chinensis* were successfully assigned in our previous study [23] by using the literature on the elution order and mass spectra, the identification process was not simple and was possible only because there was enough literature on the secondary metabolites in *S. chinensis*. For ^1^H-NMR and GC-MS, the databases from BMRB and NIST, respectively, made putative peak assignments convenient. As summarized in Table 1, nine metabolites were assigned in each plant by ^1^H-NMR analysis in this study. From the GC-MS analysis, 35 metabolites from *G. elata* (Table 2) and 29 from *R. glutinosa* (Table 3) were putatively assigned in their derivatized forms.

**Table 1:** Representative chemical shifts of metabolites putatively identified in *G. elata* and *R. glutinosa* using ^1^H-NMR spectroscopy.

*G. elata*		*R. glutinosa*	
Metabolites	Chemical shift (ppm)	Metabolites	Chemical shift (ppm)
phenylalanine *^b^*	7.38	catalpol *^a^*	6.47, 5.04
gastrodine *^a^*	7.33, 7.04	sucrose *^b^*	5.40
4-hydroxybenzyl alcohol *^a^*	7.24, 6.84	glucose *^b^*	5.23
sucrose *^b^*	5.40	glycine *^b^*	3.62
glutamate *^b^*	2.40, 2.05	arginine *^b^*	3.23, 1.71
acetate *^b^*	1.93	γ-aminobutyric acid *^b^*	3.00
alanine *^b^*	1.48	acetate *^b^*	1.93
threonine *^b^*	1.33	alanine *^b^*	1.48
valine *^b^*	1.00	threonine *^b^*	1.33

*^a^* assigned by data from literatures; *^b^* identified by BMRB database.

**Table 2:** Retention times, mass fragments, and match factors of detected metabolites in *G. elata* by GC-MS.

No.	t_R_ (min)	Identified metabolites	Mass fragments (m/z)	Match factor *^a^*
1	6.72	alanine	59, 73, 116, 147, 190	89
2	7.08	glycine	52, 73, 102, 147	91
3	8.17	disiloxane	73, 103, 131, 147, 227	83
4	9.50	valine	52, 73, 117, 144, 218	80
6	11.13	glycerol	73, 103, 117, 147, 205, 218	89
7	11.82	glycine	73, 100, 147, 174, 248	90
8	13.37	serine	73, 100, 147, 204, 218	88
9	14.05	threonine	73, 101, 117, 147, 218	84
10	16.63	malic acid	55, 73, 133, 147, 189, 233	83
11	16.75	silane	73, 147, 179, 253, 268	88
12	17.17	threitol	73, 103, 117, 147, 217	91
13	17.33	proline	73, 156, 230, 258	91
14	17.38	3,8-dioxa-2,9-disiladecane	73, 103, 147, 205, 217	89
15	17.52	aspartic acid	73, 100, 147, 218, 232	82
16	19.17	2,3,4-trihydroxybutyric acid	73, 147, 204, 220, 292	86
17	21.62	2,3,4,5-tetrahydroxypentanoicacid-1,4-lactone	73, 102, 117, 147, 189, 217	82
18	23.28	d-ribose	73, 103, 147, 217, 307	84
19	23.40	d-xylose	73, 103, 147, 217, 307	84
20	25.30	arabinitol	73, 103, 147, 217	81
21	26.57	d-glucitol	73, 103, 117, 147, 217	84
22	28.00	1,2,3-propanetricarboxylic acid	73, 147, 273	91
23	28.10	pentaric acid	73, 147, 245, 273, 319	83
24	30.63	galactose oxime	73, 103, 147, 205, 319	84
25	30.91	glucitol	73, 147, 205, 319	81
26	31.17	glucose oxime	73, 103, 147, 217	84
27	31.22	ribitol	73, 103, 147, 217	87
28	32.63	hexadecanoic acid	55, 73, 117, 145, 313	87
29	33.05	myo-inositol	73, 147, 191, 217, 318	82
30	34.22	myo-inositol	73, 147, 217, 305	87
31	35.87	9,12-octadecadienoic acid	75, 95, 117, 129, 337	92
32	36.45	octadecanoic acid	55, 73, 117, 145, 341	90
33	42.37	hexadecanoic acid	57, 73, 147, 239, 371	82
34	43.92	d-glucopyranoside	73, 103, 147, 217, 361	91
35	45.13	monostearin	57, 73, 147, 399	89

*^a^* full score at 100.

**Table 3:** Retention times, mass fragments, and match factors of detected metabolites in *R. glutinosa* by GC-MS.

No.	t_R_ (min)	Identified metabolites	Mass fragments (m/z)	Match factor *^a^*
1	7.92	propanoic aicd	73, 117, 147, 191	90
2	9.06	alanine	73, 116, 147	83
3	9.95	ethanedioic acid	73, 147, 196	90
4	12.24	l-valine	73, 144, 218	80
5	13.80	l-leucine	73, 147, 158	90
6	13.58	silanamine	73, 147, 174	83
8	13.98	3,7-dioxa-2,8-disilanonane	73, 117, 147, 205	90
9	14.65	glycine	73, 147, 174, 248	83
10	15.50	propanoic acid	73, 147, 189, 292	99
11	19.52	butanedioic acid	73, 147, 233, 245	99
12	20.10	l-proline	73, 147, 156	90
13	20.25	furosardoninA	73, 100, 147, 218, 232	99
14	22.54	*N*-trimethylsylygrutam	73, 128, 147, 218, 246	90
15	24.83	xylitol	73, 147, 205, 217, 307	91
16	25.85	l-glutamic acid	73, 147, 156, 245	96
17	27.98	d-galactose	73, 147, 191, 204, 217	95
18	28.30	fructose oxime	73, 103, 147, 217, 307	91
19	28.68	d-mannitol	73, 147, 205, 217, 319	87
20	29.06	d-sorbit	73, 147, 205, 319	91
21	29.41	galactose benzyloxime	73, 147, 205, 217, 319	90
22	29.42	d-gluctiol	73, 147, 205, 217, 319	91
23	30.43	fructose	73, 103, 217, 307	83
24	32.31	1-naphthalenepentanoic acid	47, 73, 217, 305, 318	91
25	34.38	octadecanoic acid	73, 117, 204, 341	99
26	45.95	melibiose	73, 191, 204, 217	86
27	46.40	lyxopyranose	73, 141, 191, 204, 217	86
28	48.28	maltose	73, 204, 217	90
29	52.11	sucrose	73, 117, 147, 191	81

*^a^* full score at 100.

### 2.2.Determining the Origin of G. elata and R. glutinosa Using Unsupervised Statistical Analysis

**Figure 1:**
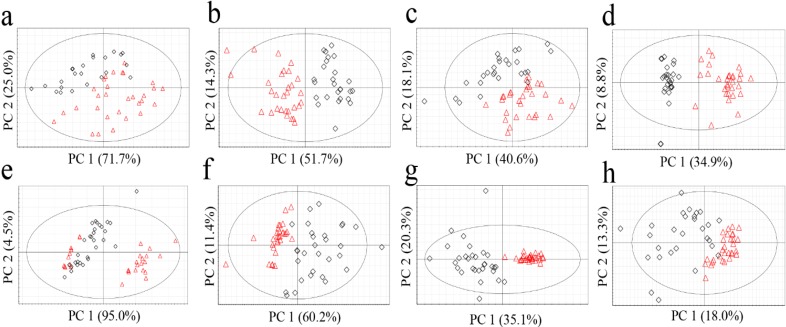
PCA score plots of *G. elata* (**a**, FT-NIR; **b**, ^1^H-NMR; **c**, GC-MS; **d**, LC-MS) and *R. glutinosa* (**e**, FT-NIR; **f**, ^1^H-NMR; **g**, GC-MS; **h**, LC-MS). Red triangles for Chinese samples; black diamonds for Korean samples.

The most popular unsupervised statistical method, PCA, was applied to the metabolomics data obtained from the four analytical methods (Figure 1). All the data tables were pre-processed with centering and scaling by unit variance, which corrected the variables by calculating the base weight of each variable as 1/standard deviation. R^2^X (cum) and Q^2^ (cum) values as the validation parameters of statistical models were displayed in Table 4.

**Table 4:** Validation of the PCA models.

Plants	Approaches	R^2^X (cum)	Q^2^ (cum)
*R. glutinosa*	FT-NIR	0.951	0.948
	^1^H-NMR	0.960	0.878
	GC-MS	0.869	0.734
	LC-MS	0.661	0.380
*G. elata*	FT-NIR	0.999	0.998
	^1^H-NMR	0.887	0.812
	GC-MS	0.795	0.697
	LC-MS	0.695	0.469

**Figure 2:**
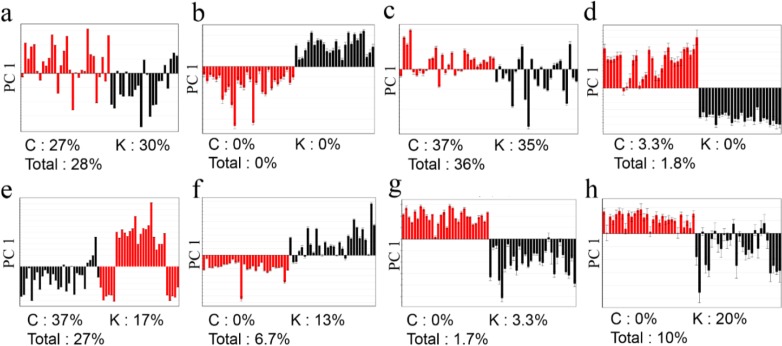
PCA column plots and misclassification rates of *G. elata* (**a**, FT-NIR; **b**, ^1^H-NMR; **c**, GC-MS; **d**, LC-MS) and *R. glutinosa* (**e**, FT-NIR; **f**, ^1^H-NMR; **g**, GC-MS; **h**, LC-MS). Red for Chinese samples; black for Korean samples.

No clear classification of the origin (Korea or China) could be found from FT-NIR data (Figure 1a,e). Classification abilities of the statistical models were evaluated as a misclassification rate. The misclassification rate was calculated from the PC1 column plots (Figure 2), given that PC1 of all the models was the key component sufficient for the origin discrimination in the two plants as in Figure 1. The resulting misclassification rates for *G. elata* were 27% (8/30) and 30% (7/23) for Chinese and Korean samples, respectively. The overall rate was 28% (15/53) (Figure 2a). The misclassification rates for *R. glutinosa* were 37% (11/30) for Chinese and 17% (5/30) for Korean samples, and the overall rate was 27% (16/60) (Figure 2e). Though FT-NIR has been reported to be useful for discriminating the quality and geographical origin of green tea [24,25], its high misclassification rates in our study suggest that analysis using FT-NIR and PCA was not useful for discriminating the origin of the two medicinal plants tested, despite its simplicity and speed.

^1^H-NMR had much lower misclassification rates for both plants than FT-NIR (Figure 2b,f). For *G. elata*, no misclassifications were found, while two misclassifications were found for Korean *R. glutinosa* (13%; 4/30), making the overall rate 6.7% (4/60). These results imply that metabolites present at concentrations high enough to be detected by a relatively insensitive technique, such as ^1^H‑NMR, can be used to authenticate their origins.

In the GC-MS analysis (Figure 2c,g), the misclassification rates were inconsistent within the two species. That is, the misclassification rates were quite high (China, 37%, 11/30; Korea, 35%, 9/26) for *G. elata*, while there was only one misclassification for *R. glutinosa* (China, 0.0%, 0/30; Korea, 3.3%, 1/30). The overall misclassification rates from the LC-MS analysis were 1.8% (1/56) for *G. elata* and 10% (6/60) for *R. glutinosa* (Figure 2d,h).

**Figure 3:**
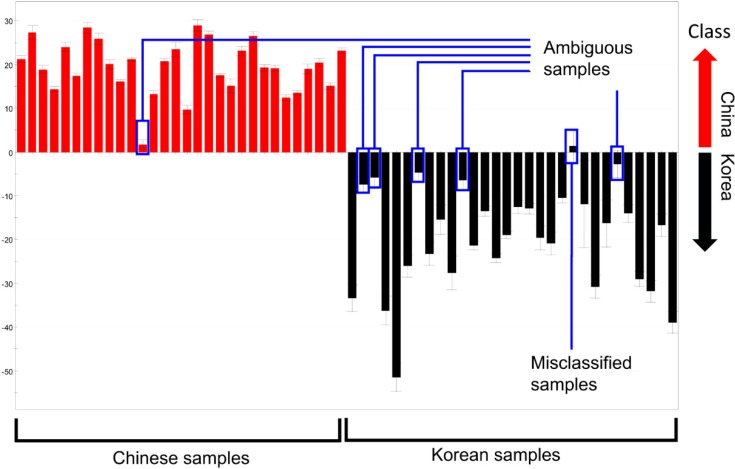
PC1 column plots of *R. glutinosa* analyzed by GC-MS (enlarged version of Figure 2g). Ambiguous samples and misclassified samples are indicated.

The distance in each discriminant model was not considered when calculating the misclassification rates above because of the inherent property of PCA. Samples of which relative distance to the maximum distance within a group was below 15% were correctly classified. Therefore, these “ambiguous” samples contributed to the low misclassification rate, despite their short distances in the discriminant model (Figure 3). For example, Chinese sample No. 12 was categorized as Chinese in the plot, but was quite close to the Korean samples, and, therefore, could introduce some uncertainty. As a result, the supervised method described below was used to further evaluate the discriminative ability of the four methods.

### 2.3.Determination of the Origin of G. elata and R. glutinosa Using Supervised Statistical Analysis

**Figure 4:**
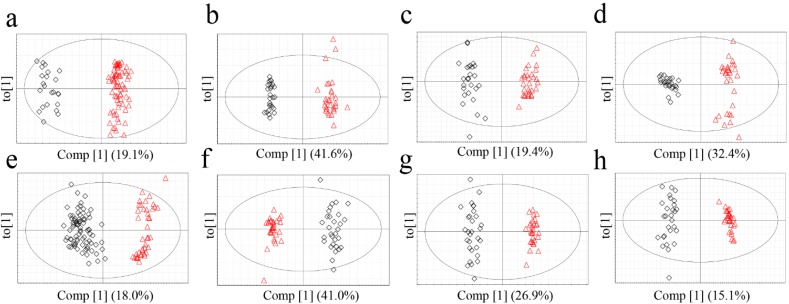
OPLS-DA score plots of *G. elata* (FT-NIR; **a**, ^1^H-NMR; **b**, GC-MS; **c**, LC-MS; **d**) and *R. glutinosa* (FT-NIR; **e**, ^1^H-NMR; **f**, GC-MS; **g**, LC-MS; **h**). Red triangles for Chinese samples; black diamonds for Korean samples.

The metabolomics data were subjected to a supervised statistical analysis method, OPLS-DA, to measure the classifications of each model (Figure 4). All of the models exhibited high-quality parameters as summarized in Table 5, with overall values of R^2^Y (cum) and Q^2^ (cum) close to 1.0. *p*‑value from cross validated-analysis of variation (CV-ANOVA) as a way of verification of model validity indicated that the models were statistically significant (Table 5) [26].

**Table 5:** Validation of the OPLS-DA models and CV-ANOVA values of each model to manifest the model’s regression.

Plants	Approaches	R^2^Y (cum)	Q^2^ (cum)	*p*-value of CV-ANOVA
*R. glutinosa*	FT-NIR	0.933	0.937	7.06 × 10^−11^
	^1^H-NMR	0.969	0.937	7.98 × 10^−28^
	GC-MS	0.971	0.904	3.33 × 10^−23^
	LC-MS	0.967	0.952	2.74 × 10^−34^
*G. elata*	FT-NIR	0.966	0.944	0
	^1^H-NMR	0.982	0.973	1.13 × 10^−36^
	GC-MS	0.969	0.924	1.11 × 10^−23^
	LC-MS	0.970	0.951	1.25 × 10^−32^

Based on Q^2^ (cum) value of the OPLS-DA models, which is indicative of classification ability of model [27], applicability of each model for origin discrimination was evaluated. The most suitable analytical techniques for discriminating between the origin of *G. elata* and *R. glutinosa* were ^1^H-NMR and LC-MS, respectively. In the analysis of *G. elata*, ^1^H-NMR had the highest discriminative ability followed by LC-MS. This result is consistent with the misclassification rates calculated from PCA, which were lowest in ^1^H-NMR (0.0%) followed by LC-MS (1.8%). These observations indicate that ^1^H-NMR and LC-MS-based approaches were appropriate for discrimination. On the other hand, for *R. glutinosa*, the Q^2^ value of the GC-MS analysis was lower than those of LC-MS and ^1^H-NMR, and GC‑MS had the lowest misclassification rate. Compared to the other techniques, GC-MS required much longer and more complicated sample preparation, including the derivatization steps, which could have lowered the reproducibility and possibly Q^2^. It is also conceivable that the unsupervised PCA performed better in analyzing the GC-MS data than the supervised OPLS-DA that is presumed to overfit the data by using class information in dimension reduction. The FT-NIR method, which had the shortest and most convenient sample preparation and analysis, was acceptable for determining the origin of *G. elata* and *R. glutinosa* based on OPLS-DA, though it was somewhat disqualified by PCA.

Upon integrating the results from the two statistical analyses, the best discrimination method differed for each plant species, which suggests that metabolomics approaches should be investigated on a case-by-case basis. Nonetheless, ^1^H-NMR and LC-MS were preferable for discriminating the origin of the two plants over the others.

## 3. Experimental Section

### 3.1. Plant Materials

The plants were collected from a number of major cultivation regions in Korea and China. A total of 26 *G. elata* samples were obtained from five cities in Korea (Chuncheon, Gimcheon, Muju, Sangju, and Asan), while 30 samples were obtained from Guangxi, Chongquing, Henan, Hunan, Shanxi, Anhui, Zhejiang, Hubei, and Guizhou in China. Thirty *R. glutinosa* samples were obtained from Andong, Jecheon, Geumsan, Seocheon, Jeongeup, and Hwasun in Korea, and 30 samples were obtained from Shanxi, Henan, and Hebei in China (Table 6). In each country, samples were collected from various farms in many cities that are relatively far from one another to represent a wide diversity of cultivation environments. The plant samples were dried for 3 days in an oven at 50 °C and ground with an electric mill (DA700, Daesung artlon, Seoul, Korea). Ground samples were sieved and particles between 125 and 300 µm were used for analysis to ensure consistent extraction efficacy among the samples.

**Table 6:** Sample collected regions of two herbal medicines in Korea and China.

*G. elata*				*R. glutinosa*			
Korea	Sample No.	China	Sample No.	Korea	Sample No.	China	Sample No.
Chuncheon	4	Guangxi	2	Andong	5	Shanxi	10
Gimcheon	9	Chongquing	1	Jecheon	5	Henan	10
Muju	3	Henan	3	Geumsan	5	Hebei	10
Sangju	1	Hunan	3	Seocheon	5		
Asan	9	Shanxi	4	Jeongeup	5		
		Anhui	3	Hwasun	5		
		Zhejiang	3				
		Hubei	10				
		Guizhou	1				

### 3.2. Near Infrared Spectroscopy

Ground samples weighing 800 mg were spread at bottom of an aluminum sample tube, and the spectrum of each sample was measured three times. For sampling, integrating sphere was employed to minimize heterogeneity and reduce deviation of baseline within data of each sample, given that it uses the diffuse reflectance. Samples were scanned from 12,000 to 4000 cm^−1^ with resolution of 8 cm^−1^ and each spectrum was obtained by averaging 32 scans using Fourier transform- near-infrared spectrometer (FT-NIR; MPA, Bruker optics, Rheinstetten, Germany). After data acquisition, data were preprocessed using multiple scattering correction (MSC).

### 3.3. Nuclear Magnetic Resonance Spectroscopy

The extraction solvent comprised 50% methanol-d_4_ (Euriso-Top, Saint-Aubin, France) in deuterium oxide (Euriso-Top) with phosphate buffer at pH 6.0 (sodium phosphate monobasic and dibasic, Sigma‑Aldrich, Madrid, Spain). 3-(Trimethylsilyl)propionic-2,2,3,3,-d_4_ acid (TMSP, Sigma-Aldrich) was added as an internal standard of chemical shift and to normalize intensity. One hundred mg of sample in 1.5 mL of extraction solvent was sonicated for 15 min at 25 °C. The extracts were centrifuged at 13,000 g for 10 min and filtered using a 0.5-µm syringe filter (Toyo Roshi Kaisha, Tokyo, Japan). The upper phase (600 µL) was transferred to an NMR tube and analyzed with a JEOL ECA 500 spectrometer, equipped with a TH5 probe (JEOL, Tokyo, Japan). The parameters were as follows: 5.7 µs (45°) pulse, 9384.0 Hz spectral width, 8 scans, and 5 s relaxation delay with 64 transients collected in 32,000 data points. The residual water spectrum was pre-saturated within the relaxation delay (4.79 ppm).

### 3.4. Liquid Chromatography-Mass Spectrometry

One hundred mg of sample was sonicated in 1 mL of methanol (J.T. Baker, NJ, USA) for 30 min at 25 °C. The extracts were centrifuged at 13,000 g for 10 min and filtered with a 0.2 µm syringe filter (Toyo Roshi Kaisha, Otawa, Tokyo, Japan). Ultra high performance liquid chromatography (ACQUITY UPLC module, Waters Corporation, Milford, MA, USA) equipped with micro-TOF QII (Bruker Daltonik GmbH, Bremen, Germany) was used to separate and detect metabolites. Metabolites were separated on an ACQUITY UPLC BEH C18 column (2.1 × 100 mm, 1.7 μm, Waters Corporation) at a flow rate of 0.2 mL/min. Elution was performed using 0.1% formic acid (Sigma-Aldrich) in water (J.T. Baker) (solvent A) and 0.1% formic acid in acetonitrile (J.T. Baker) (solvent B) at the following gradient: 0% B at 0 min, 30% B at 5 min, 70% B at 15 min, 80% B at 25 min, 100% B at 27 min, and held for 13 min. The column was pre-equilibrated with 100% A for 10 min. For ion detection, a positive mode was employed for both *R. glutinosa* and *G. elata* based on literatures reporting that the numbers of detected metabolites in the tested plants were similar in the positive and negative mode [28,29]. Electrospray ionization (ESI) was used at the following parameters: end plate offset, 500 eV; capillary voltage, 4500 eV; nebulizer, 1.2 bar; dry gas, 8.0 mL/min; capillary temperature, 200 °C; mass range, 49–1000 m/z.

### 3.5. Gas Chromatography-Mass Spectrometry

One mL of methanol was used to extract 10 mg of sample, which was sonicated for 30 min at 25 °C and filtered through a 0.5 µm syringe filter. Four hundred µL of filtered extract was purged under N_2_. After lyophilization, the extract was reconstituted in 50 µL of methoxyamine hydrochloride in pyridine (20 mg/mL, Sigma-Aldrich) and heated at 30 °C for 90 min for methoxymation of the carbonyl group. Then, 100 µL of N,O-bis(trimethylsilyl)trifluoroacetamide (BSTFA) with 1% trimethylchlorosilane (TMCS) (Sigma-Aldrich) was added and heated at 37 °C for 30 min. GC-MS (6890A connected to 5973 MSD, Agilent technologies, DE, USA) with a DB-5 column (30 m × 0.25 mm, 250 µm thickness, Agilent technologies) was used for analysis. The injection volume was 1 µL with a 1:30 split and an injection temperature of 280 °C. The carrier gas was helium at a flow rate of 0.5 mL/min. The oven temperature was as follows: initially 80 °C for 2 min, increased to 150 °C at 5 °C/min, held at 150 °C for 2 min, increased to 300 °C at 5 °C/min, and held at 300 °C for 20 min. Electron impact with 70 eV was used to ionize samples and acquire data under scan mode at 50–500 m/z.

### 3.6. Data Analysis

Peaks from the GC-MS analysis were assigned using the NIST mass spectral library [30]. ^1^H-NMR peak assignment was performed using the BMRB database (Biological Magnetic Resonance Bank) [31] and data from the literature [32,33,34].

Datasets acquired from the FT-NIR analysis were exported and binned using Unscrambler software [35]. ^1^H-NMR data were referenced to TMSP to calibrate chemical shifts (at 0.00 ppm). After calibration, the datasets were exported to ASCII format, after which 0.2–10 ppm tables were reduced to 0.04 ppm buckets. Four bins (3.29–3.37 ppm) corresponding to solvent residual peaks were removed, leaving 239 bins. ^1^H-NMR data pre-processing including peak alignment, normalization, and binning was performed with MNova (Mestrelab research, Santiago, Spain). In the LC-MS analysis, the extracted ion chromatogram of a specific m/z at a specific retention time, which corresponds to one variable in the statistical analysis, was aligned using MZmine [36] with RANSAC (RANdom SAmple Consensus) algorithm, after which the tables of aligned data were used in multivariate statistical analysis including principal component analysis (PCA) and orthogonal projection to latent structures-discriminant analysis (OPLS-DA) with SIMCA-P^+^ [37].

## 4. Conclusions

Four metabolomics approaches were applied to determine the regional origin of *G. elata* and *R. glutinosa.* Their classification abilities were evaluated based on the misclassification rates and Q^2^ values acquired from PCA and OPLS-DA, respectively. The best discrimination method differed for each plant species, *i.e.*, ^1^H-NMR and LC-MS were found as the best techniques for *G. elata* and *R. glutinosa*, respectively. Reasoned by integrating all the results from the two statistical methods, ^1^H‑NMR was generally the most prominent technique for discriminating the origins of *G. elata* and *R. glutinosa*. However, our study suggests that preliminary screening for the most suitable analytical tool and statistical method is essential to ensure the dependability of metabolomics-based discrimination of regional origins.

## Supplementary Materials

Supplementary materials can be accessed at: http://www.mdpi.com/1420-3049/19/5/6294/s1.
