# Supplementary material for: Evaluation of Four Different Analytical Tools to Determine the Regional Origin of Gastrodia elata and Rehmannia glutinosa on the Basis of Metabolomics Study
Source: Molecules. 2014 May 16;19(5):6294–308. doi: 10.3390/molecules19056294 (PMC6271526; doi:10.3390/molecules19056294)

Supplementary Materials

Figure S1. Overlaid all FT-NIR spectra of *G. elata* (**a**) and *R. glutinosa* (**b**) (not colored as their origin).

(a)

(b)

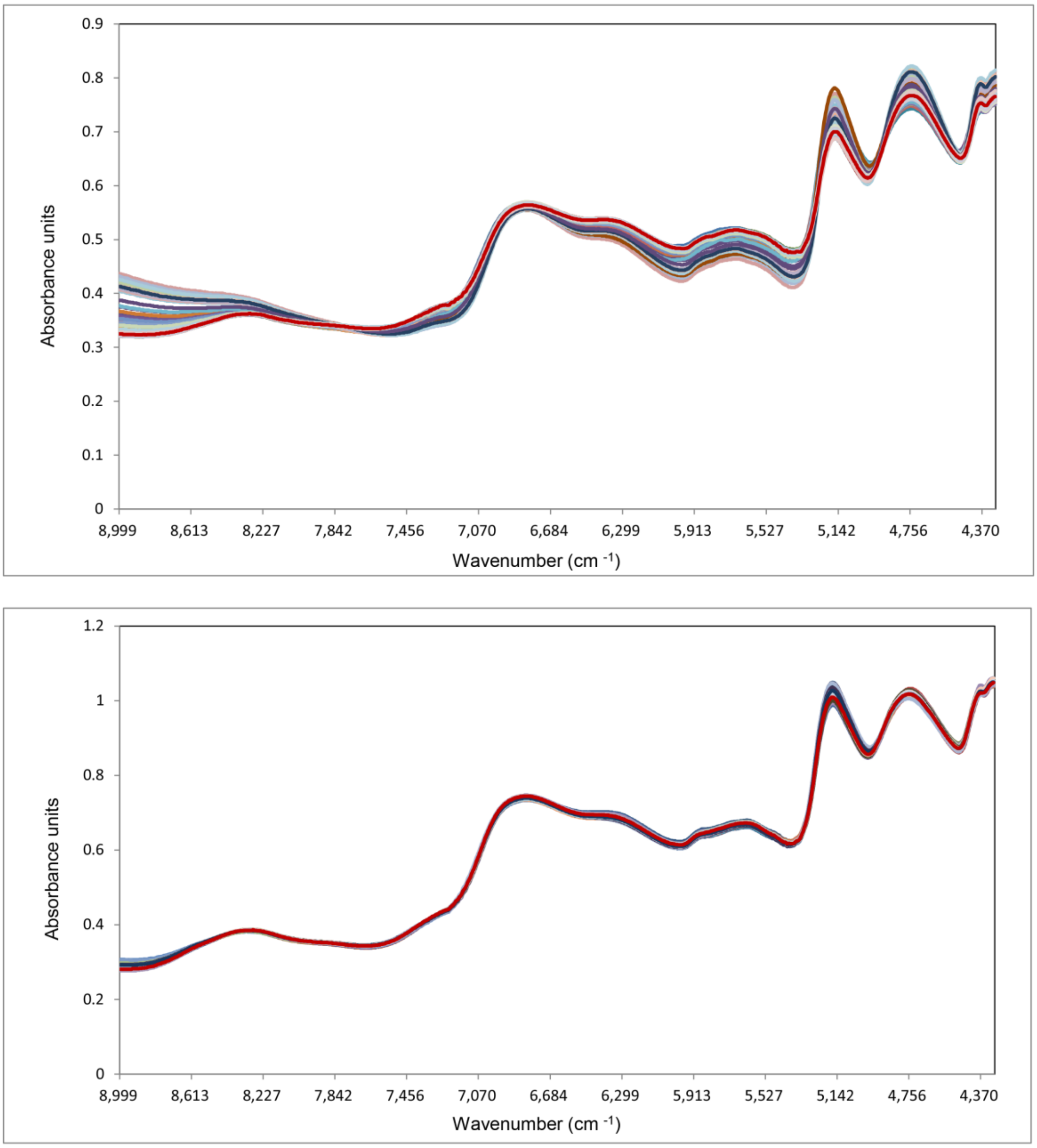

Figure S2. Overlaid all ^{1}H-NMR spectra of *G. elata* (**a**) and *R. glutinosa* (**b**).

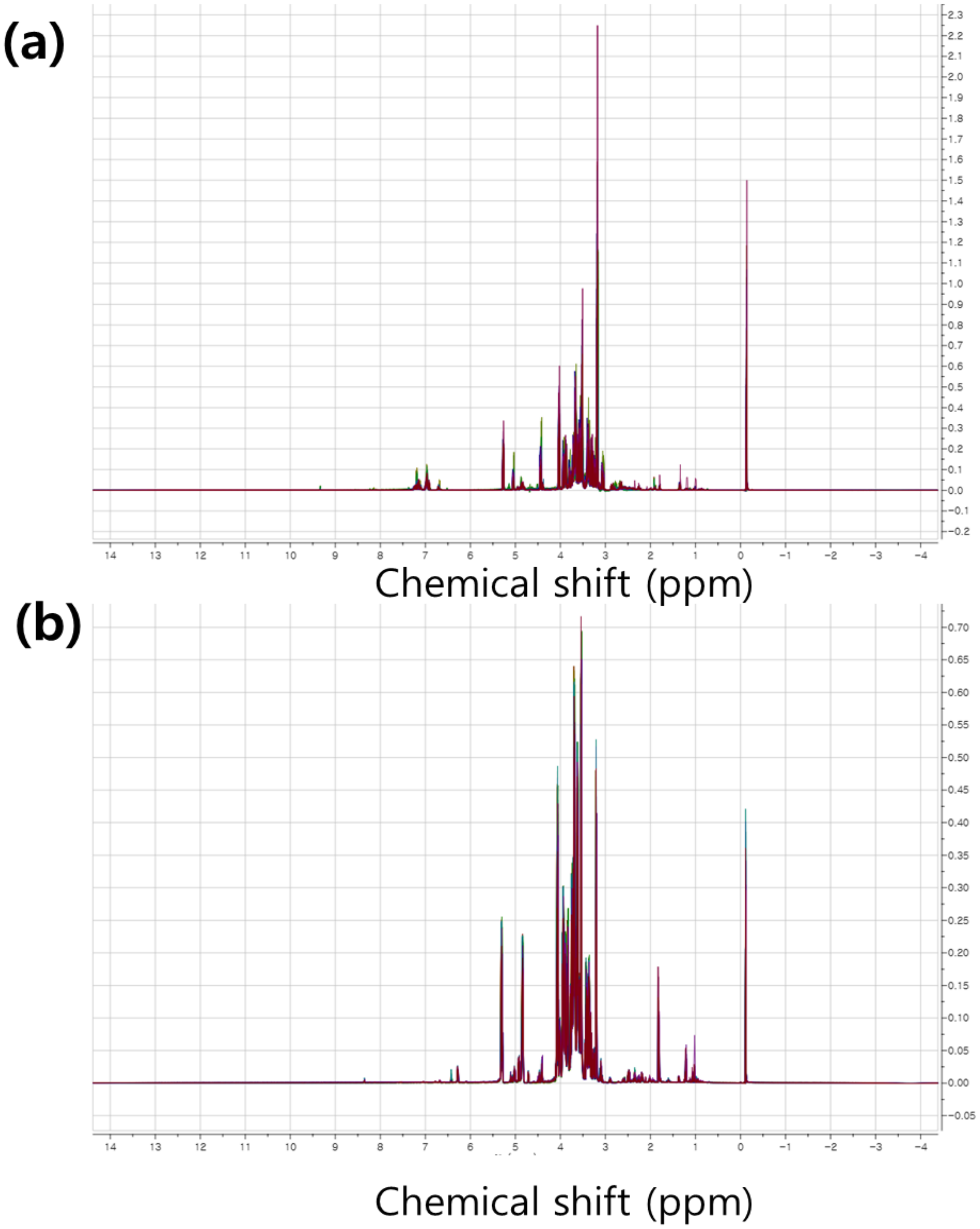

Figure S3. Overlaid all total ion chromatograms of *G. elata* (**a**) and *R. glutinosa* (**b**) acquired by LC-MS analysis.

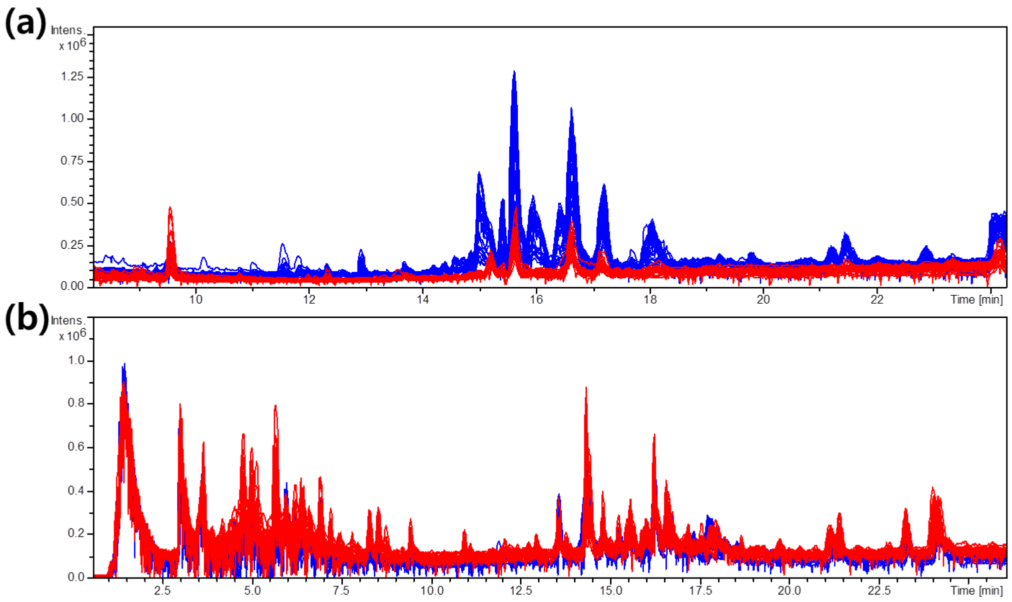

Figure S4. Overlaid all total ion chromatograms of *G. elata* (**a**) and *R. glutinosa* (**b**) acquired by GC-MS analysis.

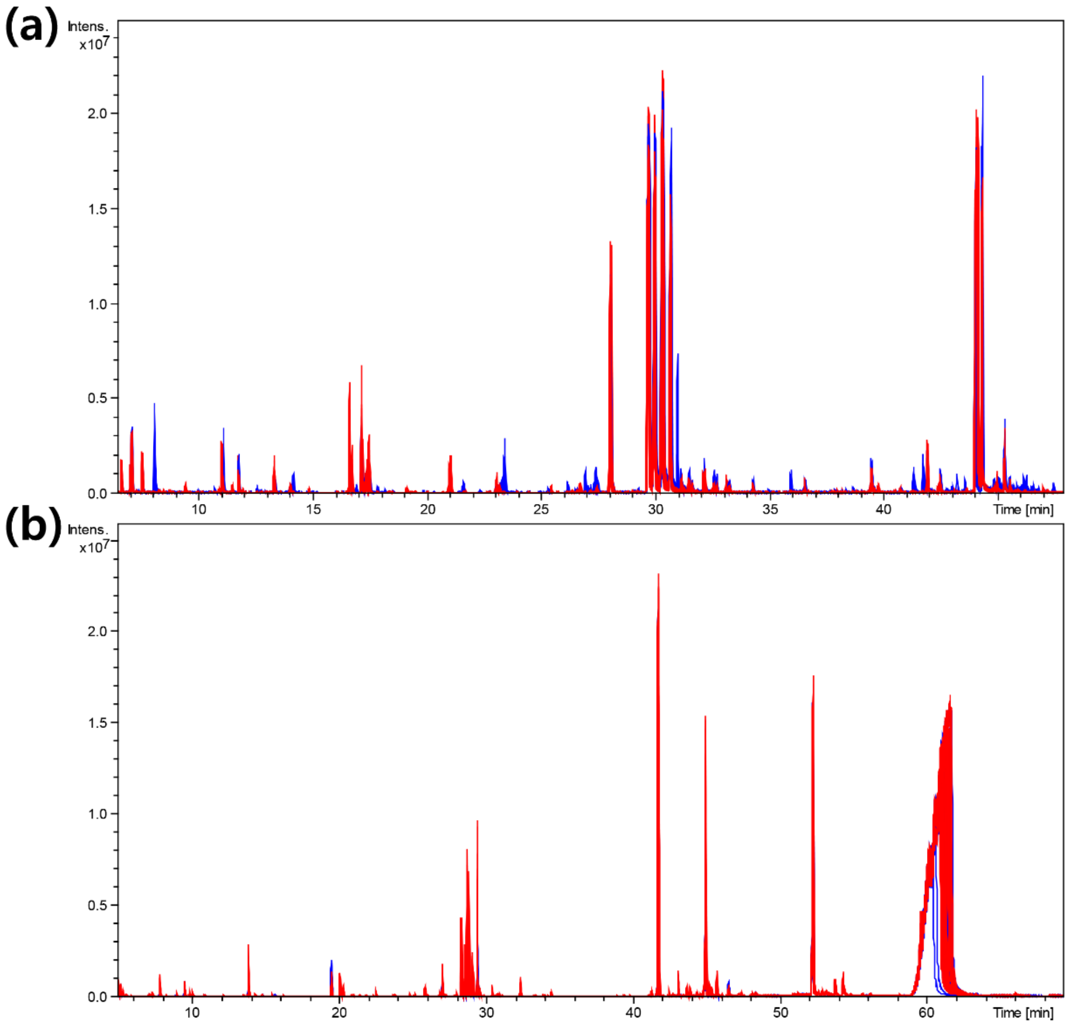

Supplement: Supplementary file 1 [file molecules-19-06294-s001.pdf]
